# Human Resources for Health in Conflict Affected Settings: A Scoping Review of Primary Peer Reviewed Publications 2016–2022

**DOI:** 10.34172/ijhpm.2023.7306

**Published:** 2023-08-05

**Authors:** Olivier Onvlee, Maryse Kok, James Buchan, Marjolein Dieleman, Mariam Hamza, Christopher Herbst

**Affiliations:** ^1^KIT Royal Tropical Institute, Amsterdam, The Netherlands; ^2^Faculty of Health, WHO Collaborating Centre, University of Technology, Sydney, NSW, Australia; ^3^World Bank, Washington, DC, USA

**Keywords:** Health Workforce, Health Policy, Health Labour Market, Health Systems, War, HRH

## Abstract

**Background:** Conflict has devastating effects on health systems, especially on healthcare workers (HCWs) working in under-resourced and hostile environments. However, little evidence is available on how policy-makers, often together with development partners, can optimize the organization of the health workforce and support HCWs to deliver accessible and trustworthy health services in conflict-affected settings (CAS).

**Methods:** A scoping review was conducted to review recent evidence (2016-2022) on human resources for health (HRH) in CAS, and critically discuss HRH challenges in these settings. Thirty-six studies were included in the review and results were presented using an adapted version of the health labour market (HLM) framework.

**Results:** Evidence from CAS highlights that conflict causes specific constraints in both the education sector and in the HLM, and deepens any existing disconnect between those sectors. Parallel and inadequate education and performance management systems, attacks on health facilities, and increased workload and stress, amongst other factors, affect HCW motivation, performance, distribution, and attrition. Short-term, narrowly focused policy-making undermines the long-term sustainability and resilience of the health workforce in CAS, and also contributes to the limited and narrow available research base.

**Conclusion:** While HRH and workforce issues in CAS include those found in many other low- and middle-income countries (LMICs), an additional set of challenges for HCWs, governance dynamics and institutional constraints in CAS ‘multiply’ negative effects on the health workforce. HRH policies, programmes and interventions must be aligned with the political and broader societal context, including the stage, severity and other dynamics of conflict. During conflict, it is important to try to monitor in- and outflow of HCWs and provide HCWs the support they need at local level or through remote measures. The post-conflict situation may present opportunities for improvement in HRH, but a clear understanding of political economy dynamics is required to better act on any such a window of opportunity.

## Background

 Globally, the number of conflict-affected settings (CAS) is rising. In 2017, the number of people living in proximity of conflict (defined as within 60 km of at least 25 conflict-related deaths) was 220 million: double compared to the number a decade ago. The Word Bank estimates that by 2030, two-thirds of the global extreme poor will be living in fragile and conflict-affected situations.^1^

 Conflict has devastating effects on health systems. It results in destruction of health infrastructure, loss of healthcare workers (HCWs), and weakening of health governance at all levels. At the same time, demand for health services increases.^2,3^ This poses a serious threat to achieving universal health coverage.

 The health workforce is the engine of any health system, as all activities and programmes have to be adopted or adapted through them.^4^ Insecurity and direct attacks lead HCWs to leave or avoid working in CAS. Consequently, small numbers of health workers are left in CAS, working in difficult conditions with often very little support.^5,6^ The COVID-19 pandemic further aggravated this situation for many HCW.

 Responding to crises in human resources of health (HRH), including in CAS, requires a systems approach.^7^ Increasing the availability of HCWs is often difficult in low-income countries and particularly in CAS. Maintaining or enhancing HCWs’ competence, responsiveness, and productivity is often also constrained, for example because of disconnection from social and professional support systems, limited supplies and equipment, increased workload and stress.^8^

 Despite the increasing availability of tools and guidelines on HRH, only a few resources are available for how policy-makers can optimize the organization of the health workforce and support HCWs to deliver accessible and trustworthy health services in CAS.^5,9^ Health systems research (including on HRH) in CAS is scarce because of lack of (financial) support, complex and rapidly changing research environments in terms of security and access, limited research capacity, difficulties in obtaining ethical clearance, mistrust towards (outside) researchers, and a lack of research application.^10,11^ Despite this, there is literature available, often based on small-scale studies in particular settings. To date, no attempt has been made to review these studies to obtain an overview of their findings and extract learnings for governments and development partners operating in CAS.

## Methods

 This scoping review aims to identify the human resources for health challenges that are specific to conflict affected settings. We thereby mean the challenges that HCWs face, constraints that governments and development partners face in managing the health workforce and its required supporting systems, and contextual factors influencing HRH. The discussion takes a broad health labour market (HLM) and health systems lens to address the complex and interconnected nature of these challenges. In anticipation of a scarcity of studies available and a variety in study designs and topics within the area of HRH we chose to conduct a scoping review. We have followed the steps outlined by Arksey and O’Malley,^12^ and completed a PRISMA-SCr (Preferred Reporting Items for Systematic reviews and Meta-Analyses extension for Scoping Reviews) checklist.

 We *identified relevant studies* reporting about HRH topics from countries on the high-intensity or medium-intensity conflict lists of the World Bank (fragile and conflict-affected situations list, financial year 2021). This criterion meant we focus on settings (countries or parts of countries) that are currently in conflict or have very recently experienced conflict. To be included, studies had to explicitly report on dimensions of conflict (eg, studies from Nigeria from non-restive areas and without reference to dynamics of conflict were excluded). Relevant HRH topics were defined as HRH policies, programmes and interventions, or factors influencing the HLM and health workers in CAS. We included only primary research studies; (systematic) reviews were excluded. The review included English language peer reviewed scientific publications published between 2016 and August 2022. Grey literature was explicitly not included to ensure the review reflected the current state of reliable, academic, peer reviewed knowledge.

*Studies were selected* using a three-pronged search strategy. First, studies from the period 2016-2019 were selected from the systematic review of Bou-Karroum et al.^5^ This systematic review comprehensively mapped the evidence base on HCWs in conflict and post-conflict settings, yet did not itself thematically analyse the content of these papers. Out of a total of 304 studies, 20 studies were included for further assessment. Second, PubMed and Google Scholar searches were conducted for papers from the period of 2016 – August 2022. These searches combined terms (in titles and abstracts) on intervention characteristics with the relevant countries (Table 1 and Supplementary file 1). Assessment of study titles (and where unclear, abstracts) resulted in 85 studies being included for further assessment. Third, 7 studies were suggested, and 3 included, through expert consultation and reviewer suggestion. Bibliographies of included studies were checked during extraction to identify additional sources.

**Table 1:** Publication Characteristics and Criteria

**Publication Characteristics**	**Keywords**
Intervention characteristics	Human resources for health, HRH, health workforce, health labour market, health [care] workers, cadre, doctors, nurses, midwives, community health workers, physicians
Countries included in the World Bank financial year 21 high-intensity or medium-intensity conflict lists	Afghanistan, Libya, Somalia, Syrian Arab Republic [Syria], Burkina Fa-so, Cameroon, CAR, Chad, DRC, Iraq, Mali, Mozambique, Myanmar, Niger, Nigeria, South Sudan, Yemen
Dimensions of conflict	Humanitarian, crisis, emergency, fragile and conflict affected [states/settings/situations/countries], post-conflict, conflict
Exclusion criteria	Lack of specific reporting on dimensions of conflict when discussing HRH issues

Abbreviations: HRH, human resources for health; DRC, Democratic Republic of Congo; CAR, Central African Republic.

**Figure 1:**
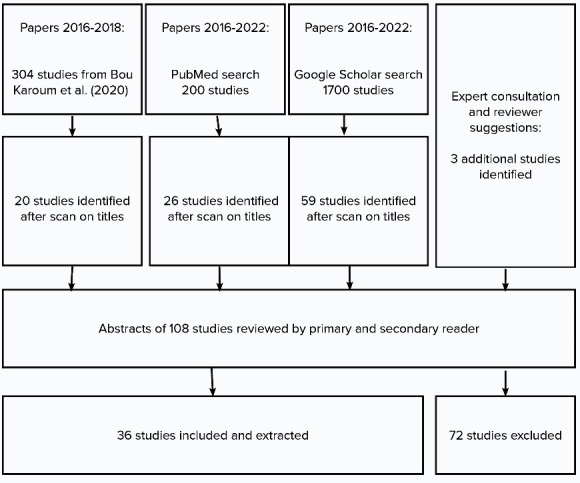
Flowchart of Study Inclusion Process.

 One hundred eight studies were divided among three researchers for abstract reading. Studies that included a focus on all three of the publication characteristics (Table 1) were included. In case of doubt, the full study was read. A second reading was done by another researcher if the article was to be included. This led to 36 studies being included in this scoping review (Figure 1).

**Figure 2:**
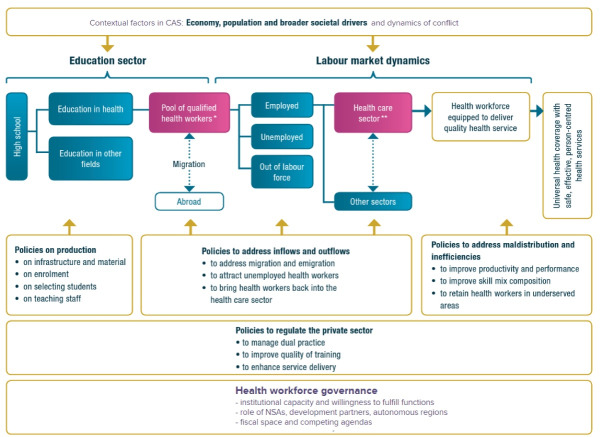
Health Labour Market Framework, Adapted to Emphasise Dynamics of Conflict and Health Workforce Governance. Abbreviations: CAS, conflict-affected settings; NSAs, non-state actors.

 Included studies were read, extracted and charted using an Excel data extraction form. We used the HLM framework as the basis for the thematic analysis of papers and presentation of results. The choice for this framework, as well as inclusion of two specific adaptations to this model to better reflect the situation in conflict affected settings are described in Box 1 and Figure 2.

**Box 1. The Health Labour Market Framework**

First developed by Sousa et al, the HLM framework takes a comprehensive view of the education sector and HLM dynamics (such as inflows and outflows of HCWs), including market failures, and links these to potential policy interventions.^13^ An important reason for us to choose this framework is its particular strength in visualizing how market disruptions within these sectors can compound issues in other parts of the framework affect workforce outcomes for years to come. This is especially relevant in CAS, as conflict causes specific disruptions across markets that further add to many difficulties already experienced in more stable LMICs.

We have chosen to further develop two elements of the HLM framework to better portray the devastating effects conflict has on the health workforce:

**Emphasis on Dynamics of Conflict**

Current versions of the HLM framework do consider ‘economy, population, and broader societal drivers’ as influencing the education sector and labour market dynamics. However, the dynamics of conflict in CAS can have such far-reaching consequences for individual HCWs and the workforce that we added this specification in the framework. In our review, it becomes clear that conflict causes significant challenges for individual HCWs (including insecurity, displacement, and mental health) as well as shocks to institutions and functioning of health systems.

**Addition of Focus on Health Workforce Governance**

While the HLM framework clearly addresses various entry points for policies, the authors felt that especially in CAS the underlying capacity and willingness of governments to fulfil key governance functions related to the health workforce cannot be taken for granted. As the review shows, in certain context the main coordinating function may fall to a non-state actor like ISIS in Syria and Iraq^14^ or ethnic health organizations in Myanmar.^15^ Moreover, in certain settings where governments are unable or unwilling to reach, development partners and/or civil society actors can take a bigger role.^16^ To highlight that an ideal stewardship role from a government cannot be presumed in CAS, we have added an additional box to highlight governance dynamics.

-----------------

Abbreviations: HRH, human resources for health; CAS, conflict-affected settings; HCWs, healthcare workers; ISIS, Islamic State of Iraq and Syria; HLM, health labour market; LMICs, low- and middle-income countries.

 Four researchers extracted relevant excerpts from the results sections of the included studies and sorted these according to key elements of the adapted HLM framework (Box 2). Quality assessment was not part of this scoping review.

**Box 2. Key Elements of the Adapted Health Labour Market Framework Used to Structure This Review**

**1. Education Sector**

The organisation, accessibility, and quality of pre-service education that affect production or supply of HCWs.

**2. Labour market dynamics**

***Inflows and Outflows***

The mobility of HCWs to enter and exit the labour market, and the ability of employing organisations to develop and implement policies that can shape and direct that mobility.

***Distribution***

The (mal)distribution of the health workforce within a country, taking into account key factors such as urban/rural, level, cadre composition, age, and gender.

***Employment and Remuneration***

HCWs’ varying employment conditions, both official and de facto, including contractual conditions and remuneration (salary, stipends, and other).

***Performance***

Various factors mediating HCWs’ (in)ability to perform, including challenges pertaining to in-service training, supervision, performance management and task divisions.

**3. Contextual Factors Affecting HCWs in CAS**

Contextual challenges particular to CAS that impact the capacity and willingness of HCWs to operate as part of the workforce, including security-related issues, economic pressures and IDP movements. Changing dynamics around gender, ethnicity, race, class, ideology and other group dynamics can impact HCWs and patients.

**4. Healthcare Workforce Governance**

The processesthat structure the roles and responsibilities of health systems stakeholders which influence if and how policies are enacted to achieve HCW outcomes (including administration of the workforce, regulation and accreditation, and policies to set and maintain strategic direction of the HCW). These processes are shaped by the interactions and relationshipsof a network of stakeholders with varying interests and power to influence governance outcomes.

-----------------

Abbreviations: HCW, healthcare worker; CAS, conflict-affected settings; IDP, internally displaced person.

###  Collating, Summarising, and Reporting Results

 Once extracted, discussions between the researchers took place on the findings for each of these main result areas of the adapted framework (see Box 2), which were summarized in narratives per results area. A first draft was discussed with key experts in HRH to further refine the focus of the results and strengthen the discussion.

## Results

###  Overview of Included Studies

 Details of the 36 included studies are shown in Table 2. The most reported contexts were Syria (n = 11), followed by the Democratic Republic of Congo (DRC) (n = 6). Most studies were qualitative (n = 29). They focused on a variety of HCWs. The findings of these studies are summarised in Table 3.

**Table 2:** Overview of Included Studies

**Author, Years and Citation**	**Country**	**Study Objective**	**Study Methods**	**Types of HCWs**	**Main Reported Elements of the Adapted HLM Framework**
Al-Areibi (2019)^17^	Libya	To examine the current Libyan medical education system, look at its positive and negative aspects, and to provide suggestions and recommendations that could help improve the quality of the system	Descriptive/opinion	Medical workforce	Education sector; HRH governance
Altare et al (2021)^18^	DRC	To identify operational challenges and investigate strategies and to maintain service delivery and quality in two health zones in North and two in South Kivu provinces in DRC in 2018	Qualitative	Representatives of private and public healthcare providers. Staff of the Ministry of Health, UN agencies, NGOs, faith-based organizations as well as HCWs (chief midwives, chief nurses, and CHWs)	Performance and motivation; inflows and outflows
Amodu et al (2021)^19^	Nigeria	To identify structural gaps influencing access to reproductive healthcare for women displaced by terrorism in Nigeria	Qualitative: critical ethnography	Primary health workers	Labour market dynamics: inflows and outflows, performance and motivation; contextual factors; HRH governance
Atia et al (2020)^20^	Libya	To assess the impacts of the accreditation process of the NCQAA on the quality of education in a private university in Libya	Qualitative: self-study report produced by the NCQAA accreditation committee during 2017-2019, using a standardized instrument	Medical students; staff working at educational institutes	HRH governance
Baba et al (2020)^21^	DRC	To identify strategies that can help to attract, support and retain midwives in the fragile and rural Ituri province	Qualitative: participatory workshop	Midwives	Education sector; labour market dynamics: inflows and outflows, performance and motivation; HRH governance
Baba et al (2020)^23^	DRC	To understand skilled birth attendants’ availability and distribution in Ituri province, North Eastern DRC from 2013 to 2017, to understand how data can be used to support evidence-informed decisions about nurses and midwives in fragile contexts	Quantitative: review of available routine data and data on local training output at provincial level	Doctors, nurses and midwives (as skilled birth attendants)	Education sector; labour market dynamics: inflows and outflows; contextual factors
Barnett-Vanes et al (2016)^23^	Iraq	To better understand the current resources and challenges facing medical schools, and the impacts of conflict on the training landscape and student experience, to provide evidence for further research and policy development	Mixed methods	Medical students; staff working at educational institutes	Education sector
Bdaiwi et al (2020)^24^	Syria	To explore current initiatives present in the north west of Syria at both the undergraduate and postgraduate level for physician and non-physician HCWs and the challenges faced in providing undergraduate education and postgraduate training during the conflict	Mixed methods: narrative review complemented with brief interviews	Allied health professionals including physiotherapists, nurses, specialist nurses, pharmacists, midwives, dentists, paramedics and emergency or anaesthetic technicians and doctors	Education sector; labour market dynamics: inflows and outflows, performance and motivation; contextual factors; HRH governance
Buny (2019)^25^	South Sudan	To explore factors contributing to staff turnover and management strategies for recruiting and retaining professional aid workers within humanitarian and development NGOs in a conflict environment in South Sudan	Qualitative: interviews and small group discussion with local and regional workers, NGO managers, expert practitioners	Expatriate humanitarian aid workers	Labour market dynamics: inflows and outflows
Elamein et al (2017)^26^	Syria	To describe a new system used mainly in areas of Syria with a substantial presence of armed opposition groups since November, 2015, to detect and verify attacks on healthcare services and describe their effect	Quantitative: based on a monitoring violence against healthcare alert network via (293-member) WhatsApp and an anonymised online data-entry tool for incident reporting	Various cadres	Contextual factors
Fardousi et al (2019)^27^	Syria	To explore health-worker perspectives on security, improving safety, managing constrained resources and handling mass casualties during besiegement in Syria	Qualitative study using semi-structured key informant interviews, conducted remotely over WhatsApp and Skype, and analysed thematically using inductive coding	Various both frontline and admin	Education sector; Performance and motivation; HRH governance
Ferdinand et al (2019)^28^	CAR	To evaluate the effectiveness of a 10-year CHW programme in CAR	Quantitative: routine case management data from CHWs and structured interviews with beneficiaries, CHWs and health facility managers	Voluntary CHWs	Labour market dynamics: performance and motivation; contextual factors; HRH governance
Footer et al (2017)^29^	Syria	To explore the complex challenges health workers face in providing care when healthcare services and personnel are themselves subjected to violence and forced to operate in the midst of ongoing human rights violations and war crimes	Qualitative: interviews with health workers	Various cadres	Labour market dynamics: inflows and outflows, performance and motivation; contextual factors; HRH governance
Fouad et al (2017)^30^	Syria	To focus on four analytical themes: attacks on health-care facilities and targeting of health workers as part of a broader pattern of systematic violations of international humanitarian law, the attrition of health workers, the challenges facing health workers in different areas, and the evolving roles of health workers	Mixed methods: scoping review, expert consultations, testimonials of health workers	Various cadres	Education sector, labour market dynamics: inflows and outflows, performance and motivation; contextual factors; HRH governance
Hamid et al (2020)^31^	Syria	To explore the impact of the provision of care of forcibly displaced Syrian MHPs to Syrian clients in the community, given shared experiences and backgrounds with clients	Qualitative: in-depth interviews with forcibly displaced Syrian MHPs across two cities in Turkey, Istanbul and Gaziantep, the latter being a city 97 km north of Aleppo, Syria	Mental health professionals	Labour market dynamics: inflows and outflows, performance and motivation; contextual factors
Ibrahem and Morgan (2022)^32^	Iraq	To explore the perspectives of healthcare professionals in Kirkuk in Iraq about the impact of the recent armed conflict (2014-2018) on their healthcare practice and training	Qualitative: Sixteen semi-structured interviews and purposive sampling of doctors and nurses who worked in conflict-affected areas in Kirkuk were selected	Doctors and nurses	Education sector; performance and motivation
Jinor (2020)^33^	DRC	To explore the lived experiences of secondary trauma for psychological assistants in the DRC	Qualitative: 13 in-depth interviews with psychological assistants	Psychological assistants, a type of lay mental health worker	Education sector; labour market dynamics: performance and motivation contextual factors
Kallström et al (2022)^34^	Syria	This research aims to determine the motivations that influence the decision of HCWs to work in a country where the conflict has been raging for a decade	Qualitative; research is based on 20 semi-structured interviews of Syrian HCWs	Various cadres	Performance and motivation, contextual factors
Kallström et al (2021)^35^	Syria	To explore the reasons why HCWs migrate from Syria, a country where conflict has been raging for over a decade	A qualitative study was performed using semi-structured interviews	Various cadres	Inflows and outflows, performance and motivation; contextual factors
Kallström et al (2021)^36^	Syria	To describe violence against healthcare from the perspective of HCWs working in Syria during – at the time of writing – the ongoing conflict	This qualitative study is based on semi-structured interviews of 25, mostly Syrian, HCWs who worked in Syria after the conflict started in 2011	Various cadres	Performance and motivation; contextual factors
Labat and Sharma (2016)^37^	DRC	To identify potential barriers to patient safety interventions from the perspective of surgical team members working in an operating theatre in Eastern DRC	Qualitative: in-depth interviews with surgical health workers in a teaching hospital	Surgical health workers, both expats and Congolese	Labour market dynamics: performance and motivation; HRH governance; contextual factors
Lar et al (2022)^38^	Nigeria	To explore the perceptions, experience, and performance of CHWs in the context of task sharing within communities in Bassa, Plateau State in Northcentral Nigeria and potentially to illuminate workforce dynamics in similar situations where regions experience conflict	Qualitative; 18 interviews with CHVs and facility heads in rural communities	CHVs	Inflows and outflows, performance and motivations; distribution
Lordfred et al (2022)^39^	DRC	This paper is a case presentation describing the process and lessons learned related to the introduction of the MISP into the first- and third-year pre-service midwifery curricula at multiple midwifery education facilities in the DRC	Qualitative; case study approach	Midwives	Performance and motivation
Lorenzetti et al (2020)^40^	Afghanistan	To evaluate a health video library intervention, a tablet-based tool to improve health promotion and counselling by CHWs	Qualitative: in-depth interviews with CHWs and CHW supervisors	Voluntary CHWs	Contextual factors
Lutwama et al (2021)^16^	South Sudan	To explore the characteristics, barriers, and facilitators to implementation of recent CHW programmes in the states supported by the HPF programme in South Sudan, in order to inform further scale-up of the BHI, and the design and implementation of CHW programmes in other low resource, CAS	Qualitative, 26 key informant interviews and a scoping review	CHWs	Performance and motivation; HRH governance
Michlig (2019)^14^	Iraq	To describe perceived changes and experiences of HCWs’ personal and professional lives in Mosul occurring during three years under ISIS	Qualitative; IDIs	Various cadres	Inflows and outflows; performance and motivation; contextual factors
Miller et al (2020)^41^	Yemen	To document the challenges to iCCM service delivery and to develop strategies for overcoming service delivery bottlenecks in conflict-affected areas of Yemen	Qualitative: in-depth interviews and focus group discussions	CHWs	Labour market dynamics: inflows and outflows, performance and motivation; contextual factors; HRH governance
Mohamed (2021)^42^	Syria	To estimate the prevalence of violence against resident doctors in Syria, investigate the association between exposure to workplace violence and health-related outcomes in terms of psychological stress, sleep quality, depression, and the overall subjective health of Syrian resident doctors, and suggest approaches to tackle this problem from the resident doctors' perspectives	Quantitative: cross-sectional survey among resident doctors	Medical doctors	Labour market dynamics: performance and motivation; contextual factors
Mowafi et al (2016)^43^	Syria	To identify the number of trauma hospitals operating in Syria and to delineate their capacities	Quantitative: nationwide survey of 94 trauma hospitals	Surgeons, nonsurgical physicians, nurses, other technical staff (both trainees and non-trainees)	Labour market dynamics: performance and motivation; contextual factors
Najafizada et al (2019)^44^	Afghanistan	To apply a multi-layered gender analysis to explore gender dynamics within the CHW system in Afghanistan	Qualitative: in-depth interviews with policy makers, health managers of NGOs implementing the programme in provinces, and CHWs and community members in villages	CHWs	Labour market dynamics: inflows and outflows, performance and motivation; contextual factors; HRH governance
Najafizada et al (2019)^45^	Afghanistan	To offer a descriptive qualitative analysis of how CHWs function as human resources for health in rural Afghanistan, and how they interact with both formal and informal health workers in the Afghan health system	Qualitative: participant observation and in-depth interviews	Voluntary CHWs	Labour market dynamics: inflows and outflows, performance and motivation; contextual factors; HRH governance
Okunogbe et al (2019)^46^	Afghanistan and South Sudan	The aim of the study is to examine the specific role of the Global Fund in strengthening HRH in the EMR. (1) What are the levels and composition of Global Fund investments in HRH in EMR countries? (2) What types of HRH activities have been supported by these investments? (3) In what ways have these investments contributed to health system strengthening in some of these countries?	Mixed methods: EMR-wide on quantitative data, qualitative case studies in Afghanistan and Sudan	Various cadres, such as: national and provincial programme officers, health management information systems officers, medical doctors, nurses, CHWs, community health supervisors, and lab technicians	Education sector; labour market dynamics: performance and motivation; HRH governance
Pare Toe and Samuelsen (2020)^47^	Burkina Faso	To explore how front-line health workers compensate for the many shortcomings they face and how the difficult working conditions affect their professional identity	Qualitative: anthropological field work, interviews with staff at dispensaries and medical centres	Physicians, assistant-doctors, nurses, assistant-nurses, midwives and laboratory technicians	Labour market dynamics: performance and motivation; contextual factors; HRH governance
Parray (2021)^48^	Afghanistan	To discuss the motivations of Afghani women to become CHWs, their status in the community and within the health system, the threatening situations under which they operate, and the challenges they face as working women in a deeply patriarchal society within a conflict zone	Qualitative; case study approach	CHWs	Performance and motivation; contextual factors
Tang and Zhao (2019)^15^	Myanmar	To audit the health systems and their performance of ethnic health organizations in selected ethnic-controlled, government-controlled or mixed-controlled areas and identify key challenges faced by the ethnic health systems in achieving universal health coverage	Qualitative: in-depth interviews and focus group discussions	Non-specific	Education sector; labour market dynamics: performance and motivation; contextual factors; HRH governance
Tappis et al (2020)^49^	Yemen	This case study examines how RMNCAH+N services have been delivered since 2015, and identifies factors influencing implementation of these services in three governorates of Yemen	Qualitative; thematic analysis and IDIs	Qualified health workers, managers and CHWs	Performance and motivation; contextual factors

Abbreviations: HRH, human resources for health; HCWs, healthcare workers; HLM, health labour market; DRC, Democratic Republic of Congo; CHW, community health worker; UN, United Nations; NCQAA, National Center for Quality Assurance and Accreditation; CAR, Central African Republic; MHPs, mental health professionals; ICCM, integrated community case management; NGO, Non-Governmental Organization; EMR, Eastern Mediterranean Region; ISIS, Islamic State of Iraq and Syria; RMNCAH+N, reproductive, maternal, new-born, child and adolescent health and nutrition; CHVs, community health volunteers; MISP, Minimum Initial Service Package for sexual and reproductive health in crisis settings; HPF, Health Pooled Fund; IDIs, in depth interviews; CAS, conflict-affected settings.

**Table 3:** Summary of Main Review Findings

**Main Elements of the Adapted HLM Framework**	**Main Review Findings**
Education sector	Conflict resulted in a higher demand for HCWs and sometimes in increased numbers of schools and establishment of new universities, which often provided sub-standard training A lack of, outdated or parallel accreditation mechanisms led to divergence in quality of services delivered by HCWs and non-recognition of trained HCWs The content of existing curricula or training could, in certain cases, be inadequate for preparing HCWs to deliver quality of care in times of conflict The quality of education was compromised by inadequate infrastructure and limited guidance and mentorship of interns
Inflows and outflows	Limited recruitment and deployment of HCWs was caused by insufficient numbers of graduates or HCWs to meet demand, and limited motivation of potential or current HCWs to work in areas with high insecurity and workload When it proved not possible to recruit specific cadres or types of HCWs, a common policy response was recruitment and deployment of CHWs Conflict often led to the (forced) outflow of HCWs, both in terms of migration to more secure areas within or outside the country and leaving the profession all together Besides insecurity, attrition was also caused by poor living and working conditions and in some cases, tension between locals, regional workers and expatriates High workload and mental health issues among HCWs caused attrition
Distribution	Maldistribution of HCWs was associated with attractiveness of working areas (urban versus rural, stable versus instable/ in conflict)
Employment and remuneration	HCWs needed and/or requested more remuneration and other benefits than they were offered There was some evidence about unequal payment of HCWs with similar functions, leading to demotivation and reduced performance
Performance	In-service training, supervision and continuous professional development opportunities were disrupted, leading to inequity in access to these job motivators among HCWs and, potentially, patient safety concerns HCWs often took on tasks and roles that they were not trained for, leading to risks for quality of care and patient and HCW safety
Contextual factors affecting HCWs in CAS	Security challenges required adjustments in the organization of and trust in healthcare, posing a challenging environment for HCWs Female health workers are more vulnerable in conflict areas Mental health impacts on HCWs can be a significant issue
HRH governance	There were significant gaps and fragmentation in leadership, HRH governance and regulation at national and regional levels Conflict can intensify corruption, which can demotivate HCWs and compromise quality of care Development partners’ short timelines of support and specific focus on certain activities or cadres, which might not be in line with government’s priorities, can disrupt HCWF composition over the medium to long term HRH policy development capacity was low, and implementation (and financing) of existing policies was constrained Non-state conflict actors, such as insurgent groups or rebel movements, are an actor group unique to CAS and bring with them serious ramifications for the health workforce A few studies reporting on Syria showed that professional associations or expatriate groups took on governance functions in non-government-controlled areas

Abbreviations: HRH, human resources for health; HCWs, healthcare workers; CHWs, community health workers; CAS, conflict-affected settings; HLM, health labour market; HCWF, healthcare workforce.

###  Education Sector

 In CAS, production or supply of HCWs coming from education and training is often disrupted. Disruptions or inefficiencies in the education pathway have adverse effects on the number, quality and recognition of (future) HCWs in CAS.

 Conflict can bring disruptions to production of HCWs as teaching staff flee, educational institutes seize operations or even become direct focus of attacks. As a response to disruptions, new institutes (often private and/or non-aligned with the government) were founded in several contexts including Libya, Syria, and DRC. However, this expansion took place in a context of limited capacity and support systems and de-facto absence of regulation or centralised planning.^17,21,24^ A few studies reported about the accreditation of (medical) education, which often lacked quality^17,23,30^ or was different in parts on the country controlled by non-state actors.^15,24^ Development partners partly and temporarily filled gaps regarding training of HCWs, but these initiatives were also not harmonized in terms of focus, content and quality of training.^24^

 Like qualified health workers, students and their teachers face serious challenges in CAS. Barnett-Vanes et al reported that students in Iraq were concerned that conflict caused them to have a low level of clinical competence due to perceived low quality of education, mental exhaustion, and fears for personal safety. The same study indicated that the majority of medical students surveyed intended to leave the country after graduation.^23^ In Syria, some medical students also had to transfer to other universities in Syria, because of the risk of arrest by the Government of Syria if they were thought to oppose the government or to have studied at ‘opposition institutions.’^24^

 Fouad et al reported that the content of existing curricula and training was inadequate for preparing HCWs to deliver quality of care in times of conflict.^27^ The medical training in Syria did not include specialisation in trauma management, intensive care, or emergency medicine, which resulted in HCWs needing to acquire these knowledge and skills on the job.^27,30^ The quality of education was compromised by inadequate infrastructure, issues with recruitment and retention of qualified professors, and limited guidance and mentorship provided to interns.Al-Areibi reported that the operation of educational facilities in Libya was highly constrained by, amongst other factors, a lack of physical space, insufficient library resources and inadequate laboratory equipment.^17^ Barnett-Vanes et al described that in Iraq staff recruitment and retention became a serious issue, resulting in missed classes, study delay and perceived lower quality.^23^ Footer et al reported that in high intensity conflict settings like in Syria, interns and other HCWs in training often had to take on more responsibilities than could normally be expected, whilst at the same time receiving less guidance, supervision and mentorship. This had implications for standards of care and caused significant psychological pressure and increased physical workload.^29^ Some adaptations to curricula and teaching methods were reported, such as the inclusion of focus on gender based violence in the midwifery curricula in the DRC^39^ and distance learning being provided by certain institutions in Syria.^24^

###  Labour Market Dynamics

####  Inflows and Outflows

 Studies describe specific several conflict related dynamics that impact the mobility of HCWs to enter and exit the labour market, and the ability of employing organisations to develop and implement policies that can shape and direct that mobility. Disconnects and imbalances between in- and out-flows of HCWs in the labour market lead to an inadequate and/or unstable pool of HCWs.

 The review found that most studies indicated limited recruitment and deployment of HCWs was caused by insufficient numbers of graduates or HCWs to meet demand. Other identified factors include the limited motivation of potential or current HCWs to work in areas with high insecurity and high workload. Lar et al described that heightened intercommunal tensions in Nigeria made communities more suspicious of community health workers (CHWs) from outside the main ethno-religious group, which narrowed the potential scope of people to be deployed.^38^ When it proved not possible to recruit specific cadres or types of HCWs, a common policy response was recruitment and deployment of CHWs. CHWs programmes can be effective when CHWs receive appropriate training, supervision and other support. However CHWs, like in many low- and middle-income countries (LMICs), were found to have poor job security and be under-remunerated.^19,48^

 Conflict often led to the (forced) outflow of HCWs, both in terms of migration or internal displacement to more secure areas within or outside the country and leaving the profession or health sector employment all together.Fear for being targeted professionally as well as general decreasing security for health workers and their families is reported on in literature and is a key driver for both of these outflows.^18,35,49^ Studies from Syria reported that many health workers either fled or voluntarily emigrated, and that whilst this attrition affected the whole of Syria, the situation was very different between government-controlled areas and nongovernment-controlled areas.^30^ Other reported factors causing attrition were economic meltdown caused by conflict^30^ and better opportunities abroad.^44,49^ A key issue to understand the volume of outflows is inadequate data monitoring HCW outflow.^22^

 Reliance on external humanitarian HCWs was noted in some studies to fill gaps. Tensions between locals, regional workers and expatriates lead to HCWs leavingIn South Sudan, HCWs reported unfavourable government policies towards foreign workers and short-term employment contracts due to stringent donor funding requirements.^25^

####  Distribution

 The review indicated that maldistribution of HCWs was associated with attractiveness of working areas (urban versus rural, stable versus unstable/in conflict). In Afghanistan, reliance on CHWs in remote and more conflict prone areas meant they were effectively the main health workers accessible to certain communities.^48^ Some studies described differences in distribution in terms of the gender of HCWs. A study in DRC found that less than one third of nurses in rural health districts were female, while in the urban health district they made up 61% of the nurses.^22^ This can be problematic, particularly in settings where certain cadres are underrepresented by one of the genders or where communities have a preference to receive care by one gender.

####  Employment and Remuneration

 The studies that reported about pay or incentives in CAS showed that HCWs often received little and irregular income, and that this was worse in rural and unstable areas than in urban and more stable areas.^21,22^ Risk allowances were officially in place in some settings like the DRC, but often remained unpaid.^21,22^ Health workers reported a need to supplement income through user fees in DRC^21^ or through over prescription of certain drugs in Myanmar.^36^ A few studies reported on issues related to staff within the same institution being employed on different contracts, terms and conditions.^21,45^ One study in Yemen described that HCWs actively shifted between contracts to find better pay – with one study specifically noting the distorting role of humanitarian organisations, who come in in offering higher pay. This leads to tensions between different workers on different types of contracts.^49^ One study focusing on experiences of frontline workers in Burkina Faso highlighted that it was generally considered an advantage to be employed in the public sector compared to the private sector, because it was perceived to be a more secure position and it included a pension system.^47^ In Syria, at the beginning of the conflict some doctors would remain officially employed and paid by the government, but volunteer in underground hospitals.^34^

 A study among local aid workers in South Sudan reported that they may be influenced to continue working in the humanitarian and development sectors, if there is increasing availability of competitive salaries and benefits such as medical insurance and paid time off, training and education opportunities, career progression, performance feedback, effective communication, and staff empowerment opportunities.^25^

####  Performance 

 This review found significant issues with performance of HCWs in CAS, with issues around in-service training, supervision, performance management and task divisions worsening labour market inefficiencies and negatively impacting retention and motivation.

 We found that in-service training, supervision and continuous professional development opportunities were disrupted in conflict settings, leading to inequity in access to these job motivators among HCWs and, potentially, patient safety concerns. In CAR, refresher training was organized only irregularly for CHWs, and major discrepancies were found between districts in relation to supervision practices.^28^ In a study of surgical teams in DRC, all interviewees highlighted the need for continuous medical education while underscoring inequity in access to training. Some participants highlighted the increased risk of errors due to the lack of ‘supervision’ of inexperienced trainees in surgical care.^37^ Non-governmental organizations (NGOs) sometimes filled the gap by providing in-service trainings, with evidence from South Sudan indicating that this led to variations in content and duration of training.^16^

 Several studies reported increased workload for HCWs remaining in position, often aggravated by the double impact of fewer colleagues to treat more and more complex cases (such as trauma cases after an attack, or increased levels of communicable diseases due to an influx of internally displaced persons [IDPs]).^30,32^ HCWs often took on tasks and roles that they were not trained for, leading to risks for quality of care and patient and HCW safety. An example was related to junior doctors being hurriedly employed in full service in Syria, and various specialists having to retrain in active conflict areas where HCWs were forced to re-specialise on triage and trauma.^27^ In Syria, insufficient numbers of HCWs led to an increased focus on roles of non-physician HCWs and skill substitution. Bdaiwi et al reported that specialised dialysis nurses in Syria had been taking the roles of renal specialists: this was done with the support of the single remaining renal physician and a team of expatriate renal physicians providing training and advice.^24^ Despite all challenges facing them, studies widely report the importance of intrinsic factors such as humanitarianism and medical ethics as a strong motivating factor for HCWs to remain in position.^29,34^

####  Contextual Factors Affecting HCWs in CAS

 This review identified many contextual challenges particular to CAS that impact the capacity and willingness of HCWs to operate as part of the workforce, including security-related issues, economic pressures and IDP movements. Changing dynamics around gender, ethnicity, race, class, ideology and other group dynamics impact HCWs and their patient relationships — with female HCWs notably facing many additional challenges in CAS.

 Insecurity of health workers, both due to targeting based on their professional status and more generally as a citizen living in a conflict zone, is tragically the most commonly reported theme throughout the literature reviewed.^18,22,25-27,29,30,32-34,36,37,41,43,44,49^ In Syria this included persecution for treating protestors,^27,30^ systematic attacks and bombings on health facilities in opposition held areas (including strategies such as chemical attacks and ‘double-tap’ attacks meant to specifically target onrushing aid personnel).^26,48^ Fouad et al interpreted the many ways in which health and HCWs become active targets of conflict actors as a pattern of ‘weaponisation of health.’^30,50^ Security challenges required adjustments in the organization of and trust in healthcare, posing a challenging environment for HCWs. In Syria, the threats to HCWs and patients were so severe that they necessitated a re-organisation of healthcare infrastructure and staff. For example, various wards of hospitals were split among different (fortified) positions, and hospitals were placed underground to reduce risks of targeting and minimise potential for all services being interrupted.^29,30,36^ Field hospitals in homes, schools, basements, mosques, and caves were established to treat casualties before transporting patients to permanent medical facilities.^30^ Mowafi et al reported on the problem of transferring patients in need of specialty care between hospitals in active war zones in Syria.^43^ Insecurity and curfews could also force HCWs to change their working hours, with evidence from the DRC indicating shorter opening hours to avoid the need for HCWs to travel home at night.^18^ In Yemen and Nigeria, offering services in areas other than CHWs’ and supervisors’ village of residence, became a problem.^38,41^ In Afghanistan, safety on the roads was a problem for particularly female CHWs.^45^ In DRC, ethnic grouping on either side of the conflict generated patient distrust of HCWs or between each other, which sometimes compromised HCWs’ attitudes towards particular ethnic groups.^37^ Furthermore, for the predominantly female lay mental health workers in DRC, gender-based violence was a fear, as well as one of the biggest reasons for secondary trauma.^33^

 The enormous stress and impact on mental health of HCWs is described in many studies.^14,29,33,36^ The stress of providing care was partly caused by the scarcity of HCWs, leading to heavy workload, an inability to “switch off” from work, and a feeling of helplessness because of the number of people they could not assist.^29^ Moreover, delayed or non-payment of salaries and increasingly expensive cost of living could complicate HCWs home situation.^14,48^ Psychological assistants (lay mental health workers) in DRC were susceptible to secondary trauma, as they may have been exposed, or were afraid to get exposed, to traumatic events their clients had experienced.^33^ Footer et al reported on the stress of health workers, after being threatened being to prioritise the government forces, at the expense of the civilian population.^29^ Provision of care to protestors was criminalized in Syria, and suspected sympathetic HCWs could be interrogated, disappeared or killed.^30,36^ In ISIS (Islamic State of Iraq and Syria) occupied Mosul (Iraq), the constant presence of morality police in clinics created a climate of fear and stress for HCWs, as even minor infractions of staff could be punished severely.^14^

####  Healthcare Workforce Governance 

 Available evidence suggests significant gaps and fragmentation in leadership and coordination, as well as institutional fragility at national and sub-national levels. In Syria, the de-facto autonomy of the Kurdish and rebel held areas spurred a fragmentation of HRH governance functions like regional healthcare workforce planning and a breakdown in coordination between decentralised structures.^24,27^ In Iraq, HCWs described the lack of flexibility of ministerial departments to adjust expectations for public health programme implementation to the realities in overstretched hospitals, resulting in tensions with patients and delays in critical care.^32^ Several studies highlighted that inter-sectoral coordination was weak. Of particular note was the often dysfunctional relationship between the Ministry of Health and the Ministry of (higher) Education,^17,45^ that have shared responsibility for health sector education and/or accreditation. In South Sudan, implementation of CHW programmes was fragmented due to lack of coordination and regulatory frameworks.^16^ In the DRC, Labat and Sharma reported on increased corruption, organized crime (where people do not dare to take action because of their own safety), a lack of accountability of hospital management towards national health authorities, and patronage in human resource management (where people supportive of authorities are at higher levels).^37^ In Nigeria, patent and proprietary medicine vendors – small owner operated drug retailers – provided services that went far beyond the role that was outlined by the pharmacy council of Nigeria.^19^ Co-ordination between the national level government and United Nations agencies and other development partners on health, including HRH, can be limited and uneven in CAS, as was the case in Nigeria.^19^ In South Sudan, a large influx of donor money in the context of inadequate funding resulted in overdependence on donors.^16^ In the most unstable (humanitarian) settings, the de-facto absence of government capacity in the health sector has created a scenario in which international non-governmental organizations (INGOs), faith-based organisations and non-profits have taken on increased responsibilities. Risk of parallel services was identified in some papers, as was donor rigidity in approach when more flexibility is required.^19,24,44,46^

 The few studies that reported on HRH policy development showed capacity was low, and implementation (and financing) of existing policies was constrained.In DRC, the Ministry of Public Health developed national strategies to attract and retain different categories of HCWs. However, many of these strategies were either not implemented or only poorly implemented as Provincial Health Offices had limited control over the recruitment and deployment of health workers, which may be partly attributed to the HRH information system being unreliable. This resulted in inequitable distribution of HCWs across the districts.^21,22^ Policy implementation gaps were also reported in a study from Burkina Faso,^47^ where implementation of a national-level policy on free prenatal consultations and provisions for obstetric emergencies was launched before the technical management and communication plan was ready. Not only did this prove problematic for attaining the policy goals, it also put HCWs in a difficult position where they were expected to deliver on the promises of the policy to patients, without the funding and resources to provide services. Amodu et al reported on low budgetary provisions for health in conflict affected areas of Northern Nigeria and highlighted a gap between budget allocation and actual release of the budget in CAS. Funding for IDP-related (health) concerns was lower than expected and insufficient to carry out the numerous activities that implementing agencies were saddled up with. The study identified links to “[broader] issues with trust, equity, and transparency in the government and health system especially where it concerns funding management.”^19^

 In CAS where non-state actors, such as insurgent groups or rebel movements, were in control, studies indicated their interest in also controlling the health workforce. Bdaiwi et al report that in Syria, both opposition-held and ISIS-held areas saw significant focus on attracting/retaining HCWs within a provisional health system to boost their legitimacy.^24^ However at the same time there are clear examples from ISIS distrusting and using harsh measures to control HCWs.^14,36^ In Myanmar’s ethnic regions ethnic health organisations (EHOs) emerged as a parallel, overlapping health systems model where national and more localised ethnic public models coexisted under a sustained cease-fire. In these regions, EHOs were the de-facto actors organising the formal health system, though other parallel structures also existed: some facilities from the central level ministry, INGOs and private providers. While the EHOs were trusted more than central ministry-led facilities, big concerns about quality issues persisted with regard to training of HCWs, poor financial protection mechanisms, resources and regulation.^15^ In Syria professional associations or expatriate groups took on governance functions in non-government-controlled areas. Bdaiwi et al highlighted the role of the Syrian Board of Medical Specialists, (a body composed of both in-country HCWs and diaspora specialists who had aligned themselves with the Syrian interim government), who actively took on roles beyond those normally taken by professional boards, eg, the coordination and certification of education programmes in the rebel held territories.^24^ The dominant presence of non-state actors in Syria, such as NGOs/donors/humanitarians/diaspora has led in some instances to a focus on empowering HCWs, enabling them to become potential advocates for peace and using healthcare as a potential avenue for peacebuilding after conflict, as well as emphasising the responsibility of governments to guarantee health worker safety.^29,30^

## Discussion

 The evidence from the different settings and countries presented in this review highlights that dynamics of conflict both worsens ‘common’ HRH challenges found in LMICs and creates a unique set of additional challenges. The combination of these factors deepens existing disconnects between the education sector and HLM, leading to further weakened HRH in CAS. Using Syria as an example, the country already experienced a number of key HRH issues prior to the civil war, including maldistribution, high turnover of skilled staff and inadequate numbers of nurses.^51^ Once the civil war erupted, these existing issues were further exacerbated by HCWs fleeing the country/region, being arrested or even killed. Direct additional effects included non-qualified HCWs shifting to trauma care, need for concealment of facilities and fragmentation of the health system into several regionalised systems.^27,34^ So while some symptoms of disconnects in the education sector and HLM bear close resemblance to those in many other LMICs, we argue that conflict ‘multiplies’ negative workforce outcomes in CAS through more, and more complex, challenges for HCWs, governance dynamics and institutional constraints. In the next sections we discuss some of the key implications of our findings for HRH policy-making in CAS.

 HRH policies, programmes and interventions are embedded in, and have an impact on, the political and broader societal context.^52,53^ Therefore, HRH policies must take into account the context, including the stage, severity and other dynamics of conflict. Depending on the dynamics of conflict, this highlights the vulnerability of the health workforce as visibly associated to the state or opposition (requiring protection), and the responsibility of governments to provide access to services to all its citizens (avoiding maldistribution of HCWs between favoured and non-favoured regions). Moreover, the effects of health workforce reforms on larger reconciliation and mid-to-long term reconstruction efforts needs to be considered. As little discussion has taken place on what HRH policy options work ‘best’ in CAS in general, or in specific CAS, feasibility assessments and a policy dialogue on how to best spend available resources are necessary first steps.

 Long-term HRH planning efforts in CAS are constrained by a lack of (financial and human) resources, instable institutions with limited capacity (and sometimes willingness) to implement, relatively strong but unpredictable donor influence, data limitations and by the uncertainties and disconnections between (systems in) geographical areas created by ongoing conflict. While not explicitly discussed in the studies included in this review, health is often not a priority of governments in CAS^54^ and countries’ constrained economic situations and increased population movements make long-term planning difficult. Filling immediate service gaps as a short-term approach to health systems relief appears to be a common trait of HRH policies and programmes in CAS. While this makes sense given the contextual challenges and urgency of the situation, it results in a lack of focus on sustaining quality of care and building and sustaining community trust. Short-term or too narrowly focused policy-making likely undermines the long-term sustainability and resilience of the health workforce, because HRH policy decisions (especially those focused on training and deploying new cadres, changing skills mix and distribution) have long-term impact and are not easily corrected.

 Many conflict-affected countries are formally ruled by contested, repressive or mistrusted governments. This in itself has major negative implications for governance, institutions, and the effective development and implementation of HRH policies and developments. In addition, it is not a simple “before and after” dichotomy. Many of these countries had governance issues before the conflict erupted (in some cases being causes of conflict). In these contexts, state withdrawal from a sector or region may actually open space and potential for innovation and improvement, for instance in terms of job descriptions, training programmes and task-shifting, where these had become ossified. One emerging issue from the literature is the potential to increase ‘value for money’ of HRH investments through strengthening financing and decision-making authority at sub-national levels to facilitate local training, recruitment, and support to HCWs.^55^

 Partly as a result of less central-level control this review found parallel systems in the education sector as well as in deployment and management of the health workforce in CAS – for instance, the lack of central planning and proliferation of commercial training institutions is likely to contribute to imbalances in the types of cadres trained and affect health workforce composition for many years. The policy implications for how governments should deal with the existence of a health workforce controlled by non-state actors are not straightforward. On the one hand, tacit support (eg, through continuation of certification or access to training) may strengthen the legitimacy of non-state actors through increased capacity to provide services to the population under their control. However on the other hand, active obstruction of HCWs undermines the obligation of the government to protect the right to health of all of its citizens. In the post-conflict period, attention should be paid to harmonizing the regulatory divergence or duplication and promoting integration those HCWs who did not have the ‘right’ qualifications or allegiance.^56^ A failure to adequately approach this may have implications for community trust in, and access to, services.

 Labour markets are dynamic in all countries and contexts, with changing patterns of HCW mobility being an ever-present factor that must be accounted for. However, in CAS, labour markets are likely to be more unstable and fractured. The evidence on HCW outflow within and out of CAS highlights the need for improved monitoring to understand variations in HCW mobility patterns between areas regarded as relatively more or less safe. In addition to geographic mobility, there can be sectoral mobility, with HCWs moving to NGO employment if this is perceived to be more attractive.^57^ A secondary issue related to outmigration is the potential to harness capacity support from HCW diaspora working in other countries.^58^ The overall understanding of the issue of internal and international flows of HRH in CAS is hampered by a lack of accurate, complete and up-to-date data on mobility patterns. This is not unique to CAS, but there is a risk of assumption, rather than analysis, directing policy focus. Individual country contexts, in terms of ease of travel and recognition of qualifications will be amongst the determining factors. In addition, the broader context of migration, its size and complexity will impact on health system and HRH demands. Somalia, for example, has been identified as a “country of origin, destination, transit and return for a large number of people moving across the Horn of Africa region and beyond,” with a 2016 report suggesting that remittances from the diaspora accounted for 35 per cent of gross domestic product in 2012, and were among the highest in the world.^59^

 The evidence emphasises the need to address retention challenges with a co-ordinated policy response, using a bundle of different policy interventions. A systematic assessment is required to pinpoint which mix of interventions is likely to be most effective at supporting staff retention. The recently updated World Health Organization (WHO) Retention guidelines outline that community support in assuring security, housing and other livelihoods can improve HCWF retention.^60^ Though little comprehensive evidence is available on this in CAS, the role of community protection to support HCWs to practice safely and remain in CAS seems important to focus on in policy development. Moreover, trust of communities in HCWs is an especially important focus for ensuring access to care^61^– despite severe challenges, civil society driven attempts to rebuild structures in places like Syria can give hope.^29^

 The evidence highlights that the combination of under-resourcing, widespread societal trauma, and the (fear for) security threats facing facilities exacts a heavy toll on HCWs, highlighting the need for investments in mental health support services.^62^ In addition, the expansion of active monitoring of security incidents involving HCWs and patients’ needs to be supported to ensure perpetrators of violence are held to account.^63^ Particular attention must be paid to HCWs from different genders, ethnicities, ages and other intersecting characteristics that may increase vulnerability to violence.^64^ Further research on this is needed.

 To enhance HCW motivation and performance, the use of technology has been progressing rapidly, accelerated by COVID-19, and needs full consideration within CAS; eg, m-health; paying staff via mobile phones, internet access as a method of keeping in touch, online continuous professional development and performance appraisal.^65-67^ However, this review shows that there is little documented recent evidence on how technology could support HCWs in CAS, which could partly be because of problems with infrastructure and connectivity.

 Limitations of available workforce data constrain monitoring, planning and policy-making. Whilst this is not unique to CAS, the disruption and fragmentation of existing HRH information systems in CAS should be addressed. Where feasible, labour market analysis, underpinned by surveys of HCWs should be undertaken.^68^ The provision of digital HRH information systems is often part of donor-led post conflict re-building, and there is a need to ensure that the choice of systems is driven by user needs and policy priorities, and that local capacity to sustain the system is considered, with an emphasis on local “user” involvement during development, and on training of local operatives.

 Finally, investments in the health workforce will not be effective if not accompanied by investments in other elements of the health system. Effective governance is required to effectively implement the best human resources for health interventions and to co-ordinate across the other health system elements. The literature on health systems recovery after conflict often highlights that the immediate post-conflict period provides a unique opportunity for the government and development partners to increase efforts to rebuild health systems within the context of larger peace building efforts. The length, nature and challenges of a specific conflict — in addition to the specifics of the process of conflict resolution — all impact the likelihood and timing of such a window of opportunity.^54^ The implication for policy and strategy development processes is that this post-conflict opportunity cannot simply be presumed, and a clear understanding of political economy dynamics is required to better identify — and increase the chances of acting on – a window of opportunity.^69^ This is also an area that needs further research.

###  Reflection on Available Evidence

 A key finding of the review is the relative lack broad based and policy relevant scientific evidence. In our sample studies, the HLM elements most reported on were related to individual performance or HCWs experiences, with governance aspects being least reported on. However, research gaps exist related to all elements of the HLM in CAS. The most important research gap is an absence of evidence taking a ‘long-term and broad-based view’ on the HLM dynamics in CAS. Most studies take a narrower entry point, and have a shorter horizon for their examination, such as a specific aspect of retention of one cadre in a localised area or region. There is a dearth of studies that look across the range of financial incentives available. Most studies take a narrow and incomplete focus, although a comprehensive approach is essential to investigate both the *causes* (at individual and system level) of the fragmentation and variability in income levels and the *consequences* of this fragmentation. In addition, the broader HLM dynamics and the connections between different labour market characteristics are usually not considered. Political economy analysis and related approaches could help to build a better understanding of power dynamics between stakeholders influencing these dynamics — and contextualise current market failures. Furthermore, studies that focus on HCWs in CAS usually focus on those workers who have stayed, not those who have left. The evidence available may therefore not fully represent considerations relevant for all HCWs, which in turn has implications for developing effective HRH policies. Moreover, there is a scarcity of evidence adequately considering gender and equity considerations in the health workforce in general,^64^ but this is even more so the case for CAS. In addition, this review did not yield evidence on the impact of COVID-19, but the implications of the pandemic on HRH in CAS are important to consider. Some initial evidence describes enormous destabilising effects of the pandemic on health services in Yemen, where a perfect storm of intensifying conflict, a cholera epidemic linked to lack of services and COVID-19 threaten systems collapse.^70^ Whether COVID-19 becomes a new dimension of fragility or a test of the resilience of a health workforce already under pressure is something which needs further exploration. Future research could also consider including (quality assessed) grey literature.

###  Limitations

 Our scoping review only considered English language, primary research peer reviewed publications — our goal was to specifically map scientific evidence but future reviews could consider further inclusion and quality assessment of grey literature. There are a wide range of CAS contexts, each presenting unique dynamics of conflict and their interaction with the socio-cultural context and labour market. This necessarily complicates relevance of findings across settings.

## Conclusion

 Against a backdrop of growing conflict across the world, this scoping review has identified progress in generating evidence on HRH aspects of health and care systems in CAS. It has also identified continued gaps in knowledge, which limit the scope to identify and implement evidence-based policies. Whilst the HRH challenges may resemble those in many other LMICs, we argue that the unique set of societal drivers of conflict, governance dynamics and institutional constraints in CAS ‘multiply’ negative affects to the health workforce. Moreover, active conflict brings a set of additional HRH challenges, including the targeting of HCWs by combatants and effects of widespread societal trauma on the mental health of HCWs. HRH policies, programmes and interventions must be aligned with the political and broader societal context if they are to be successful. The post-conflict situation may present opportunities for improvement in HRH, but a clear understanding of political economy dynamics is required to better act on any such a window of opportunity.

## Acknowledgements

 This paper is an output of the Libya Health Sector Support Grant (P163565) program between the World Bank and Libya. Christopher H. Herbst, Mohini Kak, World Bank task led the overall work program. The team would also like to thank World Bank peer reviewers, Mickey Chopra (Lead Health Specialist) and Aarushi Bhatnagar (Economist). Moreover, the team would like to thank Jo Raven and Tim Martineau from Liverpool School of Tropical Medicine (LSTM) for their critical reflections.

## Ethical issues

 Not applicable.

## Competing interests

 Authors declare that they have no competing interests.

## Funding

 This paper is an output of the Libya Health Sector Support Grant (P163565) program between the World Bank and Libya.

## Supplementary files

Supplementary file 1. Search String PubMed.
