# Supplementary material for: Human Resources for Health in Conflict Affected Settings: A Scoping Review of Primary Peer Reviewed Publications 2016–2022
Source: Int J Health Policy Manag. 2023 Aug 5;12:7306. doi: 10.34172/ijhpm.2023.7306 (PMC10590254; doi:10.34172/ijhpm.2023.7306)
Supplement: Supplementary file 1 — Search String PubMed. [file ijhpm-12-7306-s001.pdf]

Article title: Human Resources for Health in Conflict Affected Settings: A Scoping Review of Primary Peer Reviewed Publications 2016–2022

Journal name: International Journal of Health Policy and Management (IJHPM)

Authors' information: Olivier Onvlee[1]*, Maryse Kok[1], James Buchan[2], Marjolein Dieleman[1], Mariam Hamza[3], Christopher Herbst[3]

[1]KIT Royal Tropical Institute, Amsterdam, The Netherlands.

[2]Faculty of Health, WHO Collaborating Centre, University of Technology, Sydney, NSW, Australia.

[3]World Bank, Washington, DC, USA

***Correspondence to:** Olivier Onvlee, Email: o.onvlee@kit.nl

Citation: Onvlee O, Kok M, Buchan J, Dieleman M, Hamza M, Herbst C. Human resources for health in conflict affected settings: a scoping review of primary peer reviewed publications 2016–2022. Int J Health Policy Manag. 2023;12:7306.doi:10.34172/ijhpm.2023.7306

Supplementary file 1. Search String PubMed.

((((((((((((((((((human resources for health[Title/Abstract])) AND (Afghanistan[Title/Abstract])) OR (Libya[Title/Abstract])) OR (Somalia[Title/Abstract])) OR (Syria[Title/Abstract])) OR (Burkina Faso[Title/Abstract])) OR (Cameroon[Title/Abstract])) OR (Central African Republic[Title/Abstract])) OR (Chad[Title/Abstract])) OR (Democratic Republic of Congo[Title/Abstract])) OR (Iraq[Title/Abstract])) OR (Mali[Title/Abstract])) OR (Mozambique[Title/Abstract])) OR (Myanmar[Title/Abstract])) OR (Niger[Title/Abstract])) OR (Nigeria[Title/Abstract])) OR (South Sudan[Title/Abstract])) OR (Yemen[Title/Abstract])
